# Synthesis, Characterization and Biodistribution of GdF_3_:Tb^3+^@RB Nanocomposites

**DOI:** 10.3390/ma15020569

**Published:** 2022-01-13

**Authors:** Oleg E. Polozhentsev, Ilia A. Pankin, Darya V. Khodakova, Pavel V. Medvedev, Anna S. Goncharova, Aleksey Yu. Maksimov, Oleg I. Kit, Alexander V. Soldatov

**Affiliations:** 1 The Smart Materials Research Institute, Southern Federal University, 344090 Rostov-on-Don, Russia; pankin@sfedu.ru (I.A.P.); pmedvedev@sfedu.ru (P.V.M.); soldatov@sfedu.ru (A.V.S.); 2 National Medical Research Centre for Oncology of the Ministry of Health of Russia, 344037 Rostov-on-Don, Russia; hodakovadv@rnioi.ru (D.V.K.); goncharovaas@rnioi.ru (A.S.G.); maksimovay@rnioi.ru (A.Y.M.); kitoi@rnioi.ru (O.I.K.)

**Keywords:** scintillating nanoparticle, GdF_3_:Tb^3+^, organic photosensitizer, Rose Bengal, X-ray induced photodynamic therapy, computer tomography

## Abstract

Herein we report the development of a nanocomposite for X-ray-induced photodynamic therapy (X-PDT) and computed tomography (CT) based on PEG-capped GdF_3_:Tb^3+^ scintillating nanoparticles conjugated with Rose Bengal photosensitizer via electrostatic interactions. Scintillating GdF_3_:Tb^3+^ nanoparticles were synthesized by a facile and cost-effective wet chemical precipitation method. All synthesized nanoparticles had an elongated “spindle-like” clustered morphology with an orthorhombic structure. The structure, particle size, and morphology were determined by transmission electron microscopy (TEM), X-ray diffraction (XRD), and dynamic light scattering (DLS) analysis. The presence of a polyethylene glycol (PEG) coating and Rose Bengal conjugates was proved by Fourier-transform infrared spectroscopy (FTIR), thermogravimetric analysis (TG), and ultraviolet–visible (UV-vis) analysis. Upon X-ray irradiation of the colloidal PEG-capped GdF_3_:Tb^3+^–Rose Bengal nanocomposite solution, an efficient fluorescent resonant energy transfer between scintillating nanoparticles and Rose Bengal was detected. The biodistribution of the synthesized nanoparticles in mice after intravenous administration was studied by in vivo CT imaging.

## 1. Introduction

Photodynamic therapy (PDT) is a clinically approved therapeutic modality for the treatment of several types of cancer, infections, and some other diseases [1,2,3]. PDT includes three main components: a photosensitizer (PS), oxygen, and a light source [4,5,6]. Typically, PS is injected into a patient’s body and then selectively accumulates in the tumor tissue. The irradiation of the target tissue with visible or near-infrared light of a certain wavelength activates PS, which leads to the generation of reactive oxygen species (ROS). ROS play an important role in the destruction of cancer cells, where singlet oxygen ^1^O_2_ is a key cytotoxic agent in photodynamic tumor damage. The advantage of PDT in comparison with conventional treatment modalities lies in the preservation of the integrity of the functional state of the organ in which the tumor process develops, as well as the absence of a negative effect on the general condition of the patient. However, with all the advantages of PDT, its use in the fight against deep-seated and volume tumors is ineffective. Therefore, researchers are trying to increase the efficiency of PDT, especially by searching for biocompatible photosensitizers [7] and new irradiation sources. Traditional visible light used in PDT (spectral range 400–700 nm) is limited to superficial lesions, and the penetration depth of light does not exceed 1 cm. This leads to the fact that only small superficial tumors can be treated within conventional PDT. This problem can be solved by using X-rays as an irradiation source since the penetration depth of X-ray radiation reaches up to 40 cm [8,9]. However, PSs cannot directly generate ROS from X-rays. Therefore, it is necessary to use special mediators that will efficiently convert X-rays into ultraviolet or visible light with specific wavelengths to excite PS. Thus, the efficiency and applicability of PDT can be increased through the use of nanotechnology and the development of new highly active biocompatible composite nanostructures based on luminescent nanoparticles and PS for X-ray-induced photodynamic therapy (X-PDT) [10,11,12].

Rose Bengal (4,5,6,7-tetrachloro-2′,4′,5′,7′-tetraiodofluorescein disodium salt or RB) is a hydrophilic anionic type II photosensitizer that exhibits facile photocatalytic conversion of triplet oxygen (^3^O_2_) to singlet oxygen (^1^O_2_) with a high singlet oxygen quantum yield [13,14]. Rose Bengal has been clinically used for the treatment of several types of cancer and bacterial infections. The absorption spectrum of RB perfectly overlaps with the green emission band (545 nm) of Tb^3+^ doped scintillating nanoparticles (ScNPs) with effective energy transfer between ScNPs and RB [15,16,17,18].

Gadolinium trifluoride (GdF_3_) nanoparticles are excellent host materials and characterized by effective luminescence, high transparency, low phonon energy of ~4.19 kJ/mol, and high chemical and thermal stability [19,20,21,22,23]. The Gd^3+^ ions can act as intermediates, allowing energy to migrate over the Gd^3+^ sublattice and consequently facilitating the energy transfer to luminescent centers. Moreover, Gd^3+^ has magnetic properties which makes it applicable for MRI and MPI as well as large atomic number Z allowing to efficiently attenuate X-rays, thus providing a good contrast for CT visualization. Therefore, gadolinium compounds are multifunctional materials with excellent luminescence as well as magnetic properties. The doping of Tb^3+^ ions endowed the GdF_3_ nanoparticles with strong green emission peaked at 545 nm and less intense satellites at ~490, 585, and 620 nm under the excitation of both UV light and X-ray due to the electronic transitions from the excited ^5^D_4_ state to ^7^F_J_ (J = 6–3) ground states of Tb^3+^ ion [24,25,26,27]. The best X-ray- induced luminescence intensity is achieved at a Tb^3+^ dopant concentration of approximately 10–15 at% [16,17].

This work aims to develop universal nanocomposites for X-ray-induced photodynamic therapy of deep and superficial tumors based on scintillation Tb^3+^ doped GdF_3_ nanoparticles coated with a biocompatible surfactant and effectively conjugated with RB photosensitizer. At the same time, such nanocomposites can be used as a contrast agent for computed tomography (CT) and magnetic resonance imaging (MRI). A facile and cost-effective wet chemical precipitation method in an aqueous solution allowed to obtain “spindle-like” PEG-capped GdF_3_: Tb^3+^–RB nanocomposites, where RB was conjugated on the surface of the nanoparticles due to electrostatic interactions. The emission spectrum of synthesized GdF3:Tb^3+^ matches well the absorption spectrum of RB that allows obtaining efficient energy transfer between nanoparticles and RB under X-ray irradiation.

## 2. Materials and Methods

### 2.1. Materials

Gadolinium (III) chloride hexahydrate GdCl_3_·6H_2_O (99.9%) and terbium (III) chloride hexahydrate TbCl_3_·6H_2_O (99.9%), ammonium fluoride NH_4_F (98%) were purchased from Alfa Aesar (Haverhill, MA, USA). Polyethylene glycol (PEG-1500, M = 1500 g/mol), 4,5,6,7-tetrachloro-2′,4′,5′,7′-tetraiodofluorescein disodium salt (Rose Bengal (RB), Mw = 1017.64 g/mol) were purchased from Sigma-Aldrich (Burlington, MA, USA). All chemicals were used without further purification.

### 2.2. Naked and PEG-Capped GdF_3_:Tb^3+^ ScNPs Synthesis

The series of GdF_3_:Tb^3+^ (*x* = 5, 10, 15, 20%) nanoparticles were synthesized by a wet chemical precipitation method in an aqueous solution at room temperature (RT). The typical procedure for the synthesis of naked and PEG-capped GdF_3_:Tb^3+^ nanoparticles is described as follows. First, 1-*x* mmol GdCl_3_·6H_2_O and *x* mmol TbCl_3_·6H_2_O (*x* = 0.05, 0.10, 0.15, 0.20), 3 mmol ammonium fluoride (NH_4_F) were dissolved separately in 10 mL of distilled and deionized water at RT and constantly stirred on a magnetic stirrer. After complete dissolution of the rare earth salts, the aqueous solution of ammonium fluoride was added dropwise under constant stirring for 1 h at RT. During the reaction, the previously transparent solution became turbid and white, due to the precipitation of the Tb^3+^ doped gadolinium trifluoride. After particle formation, an aqueous solution of PEG-1500 was added to the solution and stirring was continued for an additional 1 h at RT. Naked GdF_3_:Tb^3+^ nanoparticles were obtained without any addition of PEG-1500. The final product (denoted as PEG@GdF_3_:Tb^3+^(*x*)) was collected by centrifugation and washed subsequently with distilled water three times. After the centrifugation, the nanoparticles were dried in an oven at 60 °C. Nominal Tb^3+^ concentrations were 5, 10, 15, 20 at%.

### 2.3. PEG@GdF_3_:Tb^3+^−RB Nanocomposite Synthesis

A colloidal aqueous solution of PEG@GdF_3_:Tb^3+^−RB nanocomposites was prepared as follows. First, stock aqueous solution of PEG@GdF_3_:Tb^3+^ (15%) nanoparticles (5 mg/mL) and photosensitizer RB (5 × 10^−4^ M) were used. Then, the mixture was stirred for 24 h at RT. RB was adsorbed on the surface of the nanoparticles due to electrostatic interactions between the positively charged surface of the PEG@GdF_3_:Tb^3+^ nanoparticles and the negatively charged RB. To remove free RB from the solution, the resulting solution was centrifuged and the residues of the organic compound were removed. The assembled nanocomposite was resuspended in distilled water.

For quantification of conjugated RB molecules’ amount, the UV-Vis spectra have been acquired for the supernatant collected after the first centrifugation cycle. The residual concentration of RB molecules was monitored as a signal decrease of the main adsorption band (ca. 548 nm) in the UV-Vis spectra and further quantified in accordance with the calibration curve reported in Supplementary Materials (see Figure S1). Finally, the amount of adsorbed RB molecules was estimated considering that the entire volume of the RB solution was available for interaction with NPs upon stirring.

### 2.4. Experimental Methods

The physicochemical characteristics of naked and PEG-capped GdF_3_:Tb^3+^ (*x* = 5, 10, 15, 20%) nanoparticles and PEG@GdF_3_:Tb^3+^(15%)–RB nanocomposites were determined using the following experimental methods. Determination of the size, shape, and morphology of nanomaterials was carried out using transmission electron microscopy (TEM) equipped with Tecnai G2 Spirit BioTWIN (FEI, Hillsboro, OR, USA). The average hydrodynamic size of nanoparticles, particle size distribution, and ζ-potential of colloidal nanoparticle solutions were determined by dynamic light scattering (DLS) on a NANO-Flex particle size analyzer (MicroTrac GmbH, Krefeld, Germany) and STABINO (ParticleMetrix Inc, Mebane, NC, USA). The crystal structure and the average crystallite size of nanoparticles were determined using X-ray diffraction (XRD) on a D2 PHASER diffractometer (Bruker Corp., Billerica, MA, USA) using CuKα (1.5406 Å) radiation at RT in the range of 20 to 80° in the 2θ scale, with a scanning speed of 0.01°/s and a step time of 0.2 s. The quantitative and qualitative chemical composition and the concentration of doping elements were estimated using a two-dimensional micro X-ray fluorescence (XRF) spectrometer M4 Tornado (Bruker Corp., Billerica, MA, USA).

The surface chemistry and binding of organic molecules and ligands to the surface of nanoparticles were studied using Fourier-transform infrared spectroscopy (FTIR) on a Vertex 70 spectrometer (Bruker Corp., Billerica, MA, USA), TG/DSC thermogravimetric analysis on a Diamond TG/DTA equipment (PerkinElmer Inc., Waltham, MA, USA), UV-Vis spectroscopy on a UV-2600 (Shimadzu Corp., Kyoto, Japan). The magnetic properties were determined on a vibrating sample magnetometer Lake Shore 7404 (LakeShore Cryotronics, Inc., Westerville, OH, USA).

X-ray-excited optical luminescence (XEOL) of nanomaterial powders and colloidal aqueous solutions were studied using a homemade setup combining RAP-90U X-ray tube with a protective Pb-cover and Cary Eclipse fluorimeter (Agilent, Selangor, Malaysia). The fluorimeter emission slit was set to 10 nm and the gate time of 0.1 s per point was utilized. The samples in a powder form were deposited on the plastic holder forming a thin layer with a thickness of 0.4 mm. The samples in solution were measured in a plastic cuvette (3ml) fixed in the dedicated plastic holder.

In vivo CT imaging was performed on a Quantum GX-2 micro-CT device (Perkin Elmer, Waltham, MA, USA). For in vivo CT measurement, intact Balb/c (ca. 3 months age, weight 34–35 g) male mice were anesthetized with 2% isoflurane (Laboratorios Karizoo, S.A., Caldes de Montbui, Spain), using a dedicated RAS-4 anesthesia device (PerkinElmer Inc., Waltham, MA, USA). Aqueous solution 310 mM of PEG-capped GdF_3_:Tb^3+^ (15%) NPs (200 µL, ca. 13.34 mg of NPs) was administrated intravenously. After the different time intervals, micro-CT images were taken with the following parameters (tube voltage = 80 kV, tube current = 90 µA, FOW restricted by the rectangle were 86 mm × 72 mm, voxel size—140 µm). Each micro-CT scan was performed within 4 min acquisition and the corresponding radiation dose was estimated as 136 mGy.

## 3. Results and Discussion

**Figure 1:**
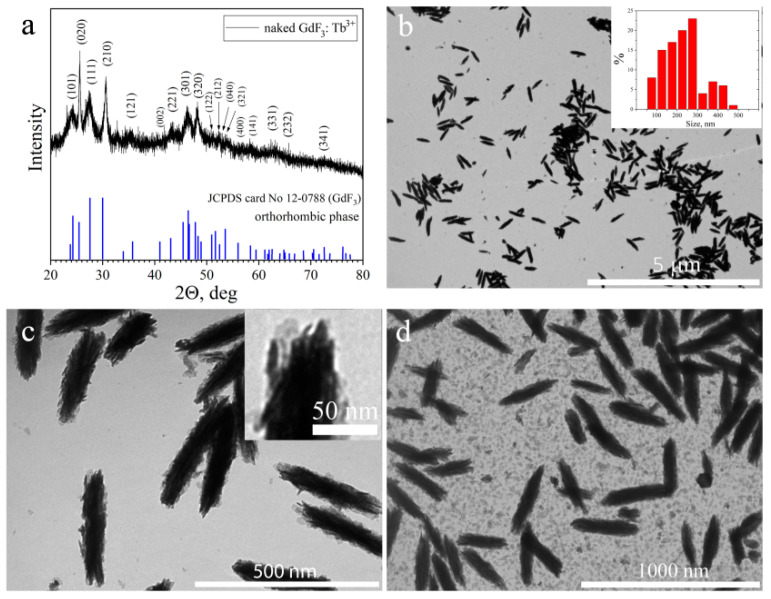
(**a**) XRD pattern of naked GdF_3_: Tb^3+^ (10%) nanoparticles with an orthorhombic structure (GdF_3_, JCPDS card No. 12-0788); (**b**,**c**) TEM images of PEG@GdF_3_: Tb^3+^ (10%) nanoparticles (insets show (**b**) the particle size in length distribution histogram and (**c**) clustered morphology of nanoparticle at high magnification), and (**d**) TEM image of PEG@GdF_3_: Tb^3+^ (10%)–RB nanocomposites.

The series of naked and PEG-capped GdF_3_:Tb^3+^ (*x* = 5, 10, 15, 20%) nanoparticles was synthesized by a wet chemical precipitation method in an aqueous solution at room temperature. Hereinafter, naked and PEG-capped GdF_3_:Tb^3+^ (10%) scintillating nanoparticles were taken as representative samples. Figure 1a shows the XRD pattern of representative naked GdF_3_:Tb^3+^ (10%) nanoparticles. The XRD patterns of all naked GdF_3_:Tb^3+^ (*x* = 5, 10, 15, 20%) nanoparticles are available in Figure S2 (Supplementary Materials). The peak positions and intensities of the XRD pattern for these nanoparticles closely match the pattern for orthorhombic GdF_3_ (JCPDS card No. 12-0788). No additional peaks of any side products were found, assuring that the samples were single phase. Based on Scherrer’s equation, the reflexes’ broadening was employed to estimate the average size of the nanocrystallites. The average crystallite size of naked and PEG-capped GdF_3_:Tb^3+^ NPs for all Tb^3+^ concentrations was ~9.2 nm. X-ray fluorescence (XRF) analysis confirmed the chemical composition Tb/Gd (5, 10, 15, 20%) of all synthesized nanoparticles, which indicates a good solubility of rare earth salts during the synthesis.

**Figure 2:**
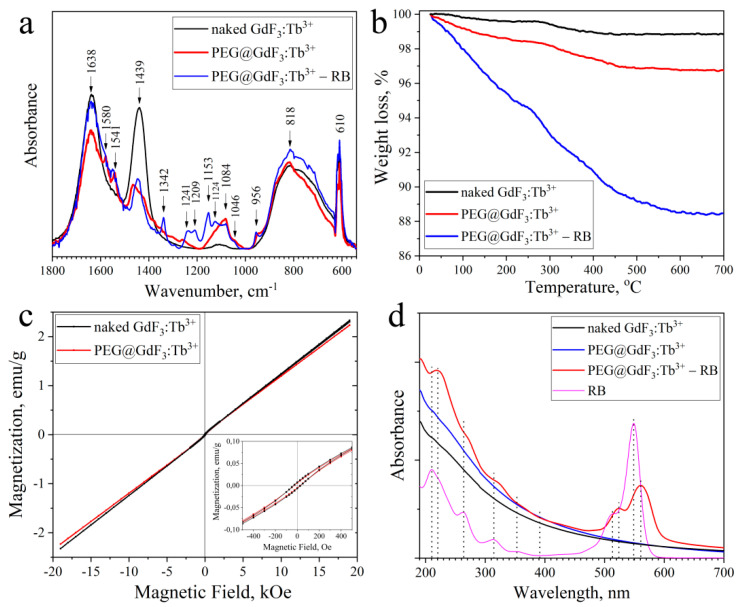
(**a**) FTIR spectra; (**b**) TG curves; (**c**) Room-temperature magnetization curves, and (**d**) UV-Vis absorption spectra of naked and PEG-capped GdF_3_:Tb^3+^(10%) nanoparticles and PEG@GdF_3_: Tb^3+^ (10%)–RB nanocomposites.

In the next stage, FTIR and TGA analyses were performed to prove PEG and RB modification of the nanoparticle surface. Figure 2a shows ATR-FTIR absorbance spectra of naked, PEG-capped GdF_3_:Tb^3+^ nanoparticles and PEG@GdF_3_:Tb^3+^-RB nanocomposites. Broad peaks in the region of 1600–1650 cm^−1^ and 650–950 cm^−1^ are associated with bending and libration modes of the adsorbed water molecules, respectively. The band at 610 cm^−1^ can be attributed to fluoride lattice, confirming the formation of the fluoride nanocrystals. For PEG-capped nanoparticles, the peaks at around 1638 cm^−1^, 1439 cm^−1^, and 1084 cm^−1^ are assigned to the methylene scissoring and C-O-C stretching vibrations of PEG [23]. The appearance of these characteristic peaks of PEG demonstrates that PEG was successfully conjugated on the surface of the nanoparticles. In the case of PEG@GdF_3_:Tb^3+^–RB nanocomposites, the peak at 1638 cm^−1^ can be assigned to C=O stretching vibration, whereas absorption peaks at 1545 cm^−1^, 1454 cm^−1^, and 1340 cm^−1^ correspond to aromatic C=C stretching modes of RB compound [28]. The band corresponding to carbonyl stretching frequency ~1700 cm^−1^ of RB has completely disappeared in the spectrum of PEG@GdF_3_:Tb^3+–^RB which means that RB molecules are attached to PEG through the carboxylic group via H-bonding and electrostatic interaction [28].

Thermogravimetric analysis of naked and PEG-capped PEG@GdF_3_:Tb^3+^ nanoparticles and PEG@GdF_3_:Tb^3+–^RB nanocomposites was probed from 25 °C to 700 °C in N_2_ atmosphere to study their thermal behavior (Figure 2b). In the TG curves of naked and PEG@GdF_3_:Tb^3+^ nanoparticles, a slight fall in weight up to 3% was observed due to the elimination of water and organic molecules. The difference observed between red and black curves is associated with additional weight loss due to the elimination of PEG molecules. The TG curve of PEG@GdF_3_:Tb^3+^–RB nanocomposites shows a decrease in the powder weight loss up to 12%, which corresponds to the weight loss of RB heated from 100 °C to 600 °C [29]. The amount of RB adsorbed on the nanoparticle surface was also evaluated by the attenuation of UV-Vis spectra of solutions obtained after mixing nanoparticles with RB molecules and stirring for a certain time (t = 10 min, up to 24 h) followed by centrifugation and removal of PEG@GdF_3_:Tb^3+^–RB precipitates from solutions (see Figure S3). It was found that for 24 h the amount of RB molecules adsorbed on the NPs surface is about 8.8 % wt., which is in good agreement with the data obtained from TG analysis (Figure 2b).

The magnetic properties of the naked and PEG-capped GdF_3_:Tb^3+^ NPs were evaluated by a vibrating sample magnetometer in the fields between [−20, +20] kOe, measured at RT. The magnetization curves are shown in Figure 2c. From the hysteresis loops of the samples, we can see that the samples exhibit paramagnetism. However, at low values of the magnetic field, nonlinear behavior of the magnetization is observed, which corresponds to a weak ferromagnetic ordering. The magnetization (Ms) of NPs is ~2.3 emu/g. The lower magnetization can be attributed to the existence of PEG, which reduces the overall magnetic moment. The inset of Figure 2c shows the region of magnification at low magnetic fields. It is observed that the value of remanence is 0.0068 emu/g and the coercive force is 31.68 Oe.

The UV-Vis spectra of naked and PEG-capped GdF_3_:Tb^3+^ nanoparticles and PEG@GdF_3_:Tb^3+^-RB nanocomposites are shown in Figure 2d. One can see that the nanoparticles are transparent to visible light and absorb UV light. The UV-Vis spectra of naked and PEG-capped GdF_3_:Tb^3+^ NPs exhibit wide absorption peaks around 215 nm and 245 nm, which can be associated with the host transition from the ground state ^8^S_7/2_ of Gd^3+^ to ^6^I_7/2_ and ^6^P_7/2_. The characteristic peak of RB at 550 nm is observed in the absorption spectrum of PEG@GdF_3_:Tb^3+^–RB nanocomposites confirming the presence of RB on the nanocomposites. Bathochromic shifts (~10 nm) of UV-Vis absorption bands of RB molecules adsorbed on the surface of GdF_3_:Tb^3+^ nanoparticles due to a change in environmental conditions are observed, which indicates the interaction of nanoparticles with RB molecules. UV-Vis absorbance spectra of RB stock solution and supernatants collected after PEG@GdF_3_:Tb^3+^ (10%) wet impregnation with a varied time of stirring or NPs’ concentration are shown in Figure S1.

X-ray-excited optical luminescence (XEOL) spectrum of PEG@GdF_3_:Tb^3+^ (10%) was collected with the following parameters of X-ray tube (U = 35 kV; I = 1.6 mA) and demonstrates characteristic radioluminescence (RL) peaks of Tb^3+^ ions at 490 nm, 545 nm, 585 nm, and 620 nm due to electronic transitions from the excited state of ^5^D_4_ to the ground states of ^7^F_J_ (J = 6–3) with the maximum intensity in the green region. The absorption spectrum of RB matches well with the green emission band (545 nm) of PEG@GdF_3_:Tb^3+^ nanoparticles. The XEOL spectra of unmodified nanoparticles GdF_3_:Tb^3+^ with different concentrations (*x* = 5, 10, 15, 20%) are available in Figure S4.

**Figure 3:**
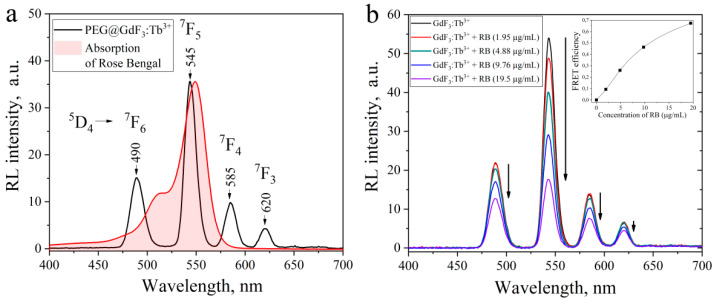
(**a**) X-ray-excited optical luminescence (XEOL) spectrum of PEG@GdF_3_:Tb^3+^ (10%) nanoparticles excited by X-ray irradiation (35 kV, 16 mA); (**b**) A decrease of XEOL intensity of colloidal PEG@GdF_3_:Tb^3+^ (10%)–RB nanocomposite solution with the amount of RB conjugated on the NPs’ surface through the fluorescence resonance energy transfer (FRET) process.

As shown in Figure 3b, the adsorption of RB molecules at the surface of PEG@GdF_3_:Tb^3+^ (10%) causes a remarkable decrease of PEG@GdF_3_:Tb^3+^ (10%) XEOL intensity that can indicate the fluorescence resonance energy transfer (FRET) process in the PEG@GdF_3_:Tb^3+^–RB nanocomposites. The fluorescence quenching of ScNPs became more remarkable upon increasing the amount of conjugated RB molecules (1.95–19.5 µg/mL of RB), which means efficient FRET between ScNPs and RB occurred. The FRET efficiency can be calculated according to the following Equation [16]:

$$E=1-\frac{F_{B}}{F_{A}}$$

where *F_A_* and *F_B_* are, respectively, the corresponding radioluminescence intensities of the GdF_3_:Tb^3+^ ScNps and GdF_3_:Tb^3+^–RB nanocomposites with different RB concentrations. The inset in Figure 3b shows FRET efficiency increased successively with the RB concentration (0–19.5 µg/mL of RB). The FRET efficiency can reach 0.67 at a high RB concentration, which indicates that RB molecules can be considered as effective acceptors for GdF_3_:Tb^3+^ ScNPs.

**Figure 4:**
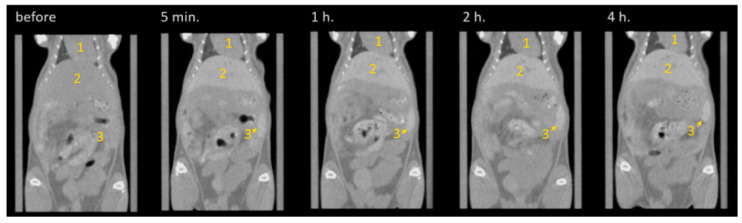
CT images taken before (left) and with different intervals after injection of PEG-capped GdF_3_:Tb^3+^ NPs. Different organs are denoted in the images: 1—heart, 2—liver, 3—spleen; the kidney is not visible within the selected 2D slice. All the rest of the structures observed in the abdominal area represent the organs of the gastrointestinal tract and bladder, which provide notable contrast also before NPs’ administration (see left panel—“before”).

In the next stage, the biodistribution of the PEG-capped scintillating NPs was accessed through in vivo CT imaging of the balb/c mice after the intravenous injection of 200 µL NPs’ aqueous solution (total dosage of Gd ca. 10 mg). Visualization before the injection and a series of subsequent scans taken at different time intervals after injection are reported in Figure 4. From the visual examination, that just after NPs’ administration (i.e., for 5 min scan), significant contrast enhancement for the liver and spleen is observed. Indeed, the borders of the liver are well-defined, and the localization of the spleen is clear compared with the pre-injection scan. Comparison of the CT scans taken after 1 h demonstrate continuous contrast increase of liver and spleen, while there is no further notable modification observed between the scans taken at 2 h and 4 h after NPs’ administration.

**Figure 5:**
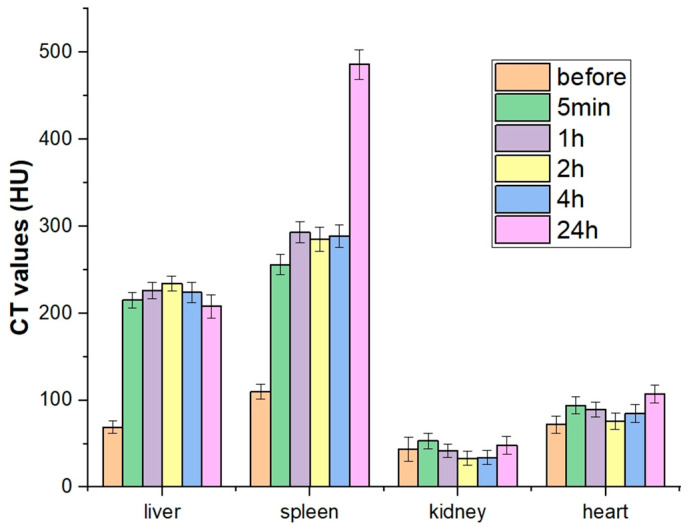
Time evolution of HU values registered for different mice organs after the intravenous injection of PEG@GdF_3_:Tb^3+^ NPs (HU values before injection reported for the sake of comparison).

In addition, NPs’ biodistribution was quantitatively estimated by analyzing the contrast for ROI, which corresponds to different mice organs (liver, spleen, kidney, and heart were taken into account). The obtained results confirmed visually observed trends (see Figure 5). In more detail, for the pre-injection, typical HU values were obtained for all organs analyzed. Just after injection (5 min), a notable contrast jump has been observed for all organs with exception of the kidney, which remains at contrast level almost invariably for the entire experiment. The heart is also characterized by a very small, but detectable increase of HU after injection. The most paramount variation has been detected for the liver and spleen, which demonstrated a sharp increase in HU from ca. 70 to ca. 215 (liver) and from ca. 105 to ca. 255 (spleen). For the next few hours after injection, a very smooth evolution of contrast ability of liver and spleen have been detected. Interestingly, for a longer time probed in our experiment (24 h), an opposite trend has been observed: the NPs slowly begin to be excreted from the liver showing a small but notable decrease in its contrast, while they continue accumulating in the spleen, leading to a further sharp contrast increase up to ca. 475 (HU).

Overall, the observed NPs’ accumulation in the liver and spleen is typical, since both organs are characterized by a well-developed vascular system, while the prolonged effect of NPs’ accumulation in the spleen can be considered as a negative effect. This will be the subject of a more detailed biodistribution study and in vivo cytotoxicity tests.

## 4. Conclusions

In summary, a facile and cost-effective wet chemical precipitation method for the preparation of PEG-capped GdF_3_:Tb^3+^ scintillating nanoparticles was proposed. The obtained nanoparticles and nanocomposites exhibited an elongated “spindle-like” clustered morphology and an average particle size of ~250 nm in length and ~60 nm in width with an orthorhombic structure. These nanocomposites can be potentially applied for X-ray- induced photodynamic therapy (X-PDT) and computed tomography (CT) imaging. Upon X-ray irradiation of the colloidal PEG-capped GdF_3_:Tb^3+^–Rose Bengal nanocomposite solution, an efficient fluorescent resonant energy transfer between scintillating nanoparticles and Rose Bengal was detected. NPs’ biodistribution in mice was quantitatively estimated by in vivo CT imaging, and significant contrast enhancement for liver and spleen was observed. However, the prolonged effect of NPs’ accumulation in the liver and spleen can be considered as a negative effect.

## Data Availability

The data reported in this work might be available upon request.

## Supplementary Materials

The following are available online at https://www.mdpi.com/article/10.3390/ma15020569/s1. Figure S1. UV-vis absorbance spectra of RB stock solution (black curve) and supernatants collected after PEG@GdF_3_:Tb^3+^(10%) wet impregnation with a varied time of stirring, NPs concentration or ultrasound (US) exposure (for 30 min).; Figure S2. XRD patterns collected for GdF_3_:Tb^3+^ (*x*%) nanoparticles with different amount of Tb^3+^ (P1- Tb5%, P2 – Tb10%, P3 – Tb15%, P4 – Tb20%).; Figure S3. The hydrodynamic radius of naked and PEG-capped GdF_3_:Tb^3+^ nanoparticles and PEG@GdF_3_:Tb^3+^ - RB nanocomposites.; Figure S4. (a) Radioluminescence spectra of PEG@GdF_3_:Tb^3+^(x=5, 10, 15, 20%) nanoparticles excited by X-rays (35kV, 16mA). The main peaks localized at 490 nm, 544 nm, 585 nm and 620 nm, corresponding to electronic transitions from the excited states ^5^D_4_ to the ground states ^7^F_J_ (J = 6–3), (b) Dependence of the peak intensity at 545 nm of radioluminescence spectra of PEG@GdF_3_: Tb^3+^ nanoparticles with different Tb^3+^ ion concentrations.
