# Supplementary material for: Synthesis, Characterization and Biodistribution of GdF3:Tb3+@RB Nanocomposites
Source: Materials (Basel). 2022 Jan 13;15(2):569. doi: 10.3390/ma15020569 (PMC8779613; doi:10.3390/ma15020569)
Supplement: Supplementary file 1 [file materials-15-00569-s001.zip › materials-1509008-supplementary.pdf]

Supplementary materials

Synthesis, characterization and biodistribution of $GdF_3:Tb^{3+}$@RB nanocomposites

Authors: Oleg E. Polozhentsev [1,*], Ilia A. Pankin [1], Darya V. Khodakova [2], Pavel V. Medvedev [1], Anna S. Goncharova [2], Aleksey Yu. Maksimov [2], Oleg I. Kit [2] and Alexander V. Soldatov [1],

[1] The Smart Materials Research Institute, Southern Federal University, 344090, Russian Federation, Rostov region, Rostov-on-Don, 178/24 Sladkova str.; nano@sfedu.ru

[2] National Medical Research Centre for Oncology of the Ministry of Health of Russia, 344037, Russian Federation, Rostov region, Rostov-on-Don, 14 liniya str., 63; rnioi@rnioi.ru

* Correspondence: oepolozhentsev@sfedu.ru; Polozhentsev O.E.

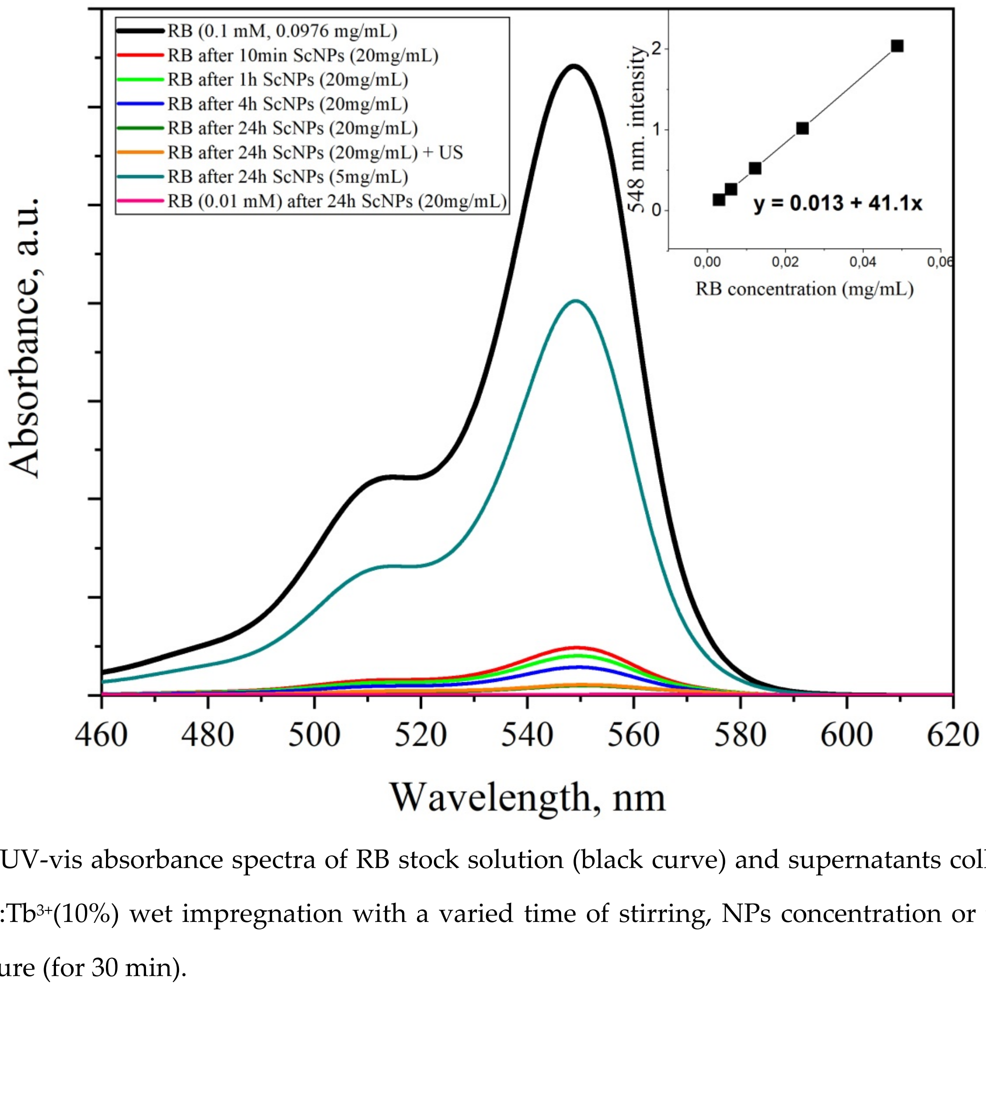

Figure S1. UV-vis absorbance spectra of RB stock solution (black curve) and supernatants collected after PEG@$GdF_3:Tb^{3+}$(10%) wet impregnation with a varied time of stirring, NPs concentration or ultrasound (US) exposure (for 30 min).

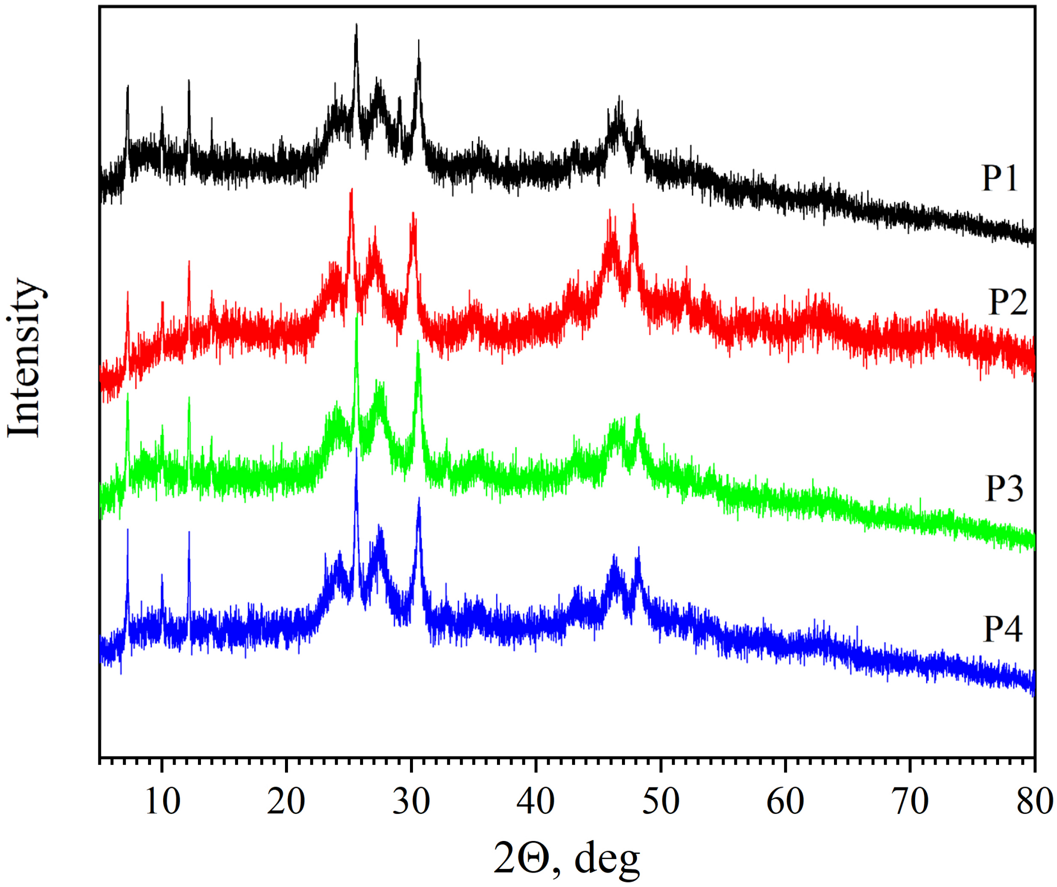

Figure S2. XRD patterns collected for $GdF_3:Tb^{3+}$ (*x*%) nanoparticles with different amount of Tb^{3+} (P1-Tb5%, P2 – Tb10%, P3 – Tb15%, P4 – Tb20%).

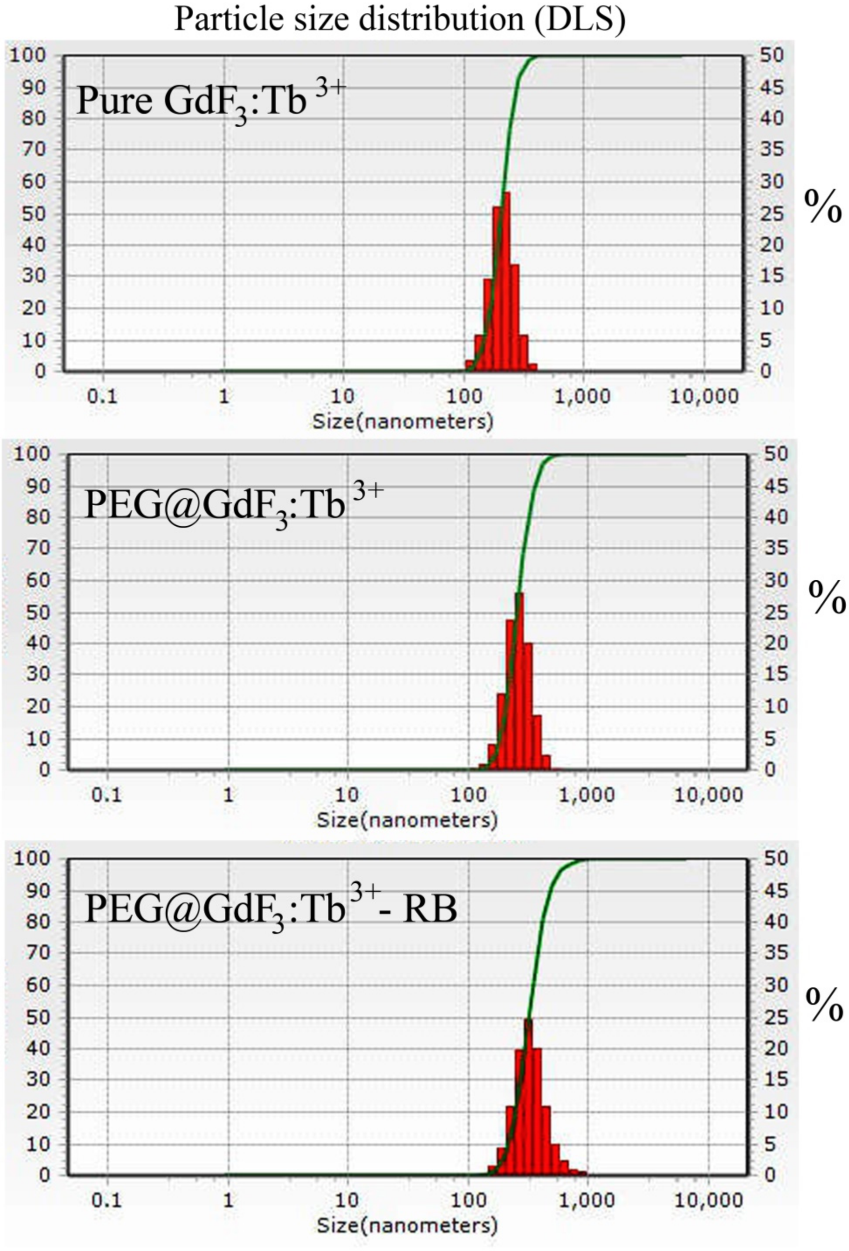

Figure S3. The hydrodynamic radius of naked and PEG-capped $GdF_3:Tb^{3+}$ nanoparticles and $PEG@GdF_3:Tb^{3+}$ - RB nanocomposites.

According to DLS measurements, the hydrodynamic radius of pure $GdF_3:Tb^{3+}$ nanoparticles is 206.5 ± 97.2 nm (polydispersity index, PDI = 0.105), 257.2 ± 124.5 nm (PDI = 0.174) for $PEG@GdF_3:Tb^{3+}$ nanoparticles, and 318 ± 184.9 nm (PDI = 0.201) for $PEG@GdF_3:Tb^{3+}$ - RB nanocomposite. A charge density (ζ-potential) for all obtained nanoparticles is +24 mV.

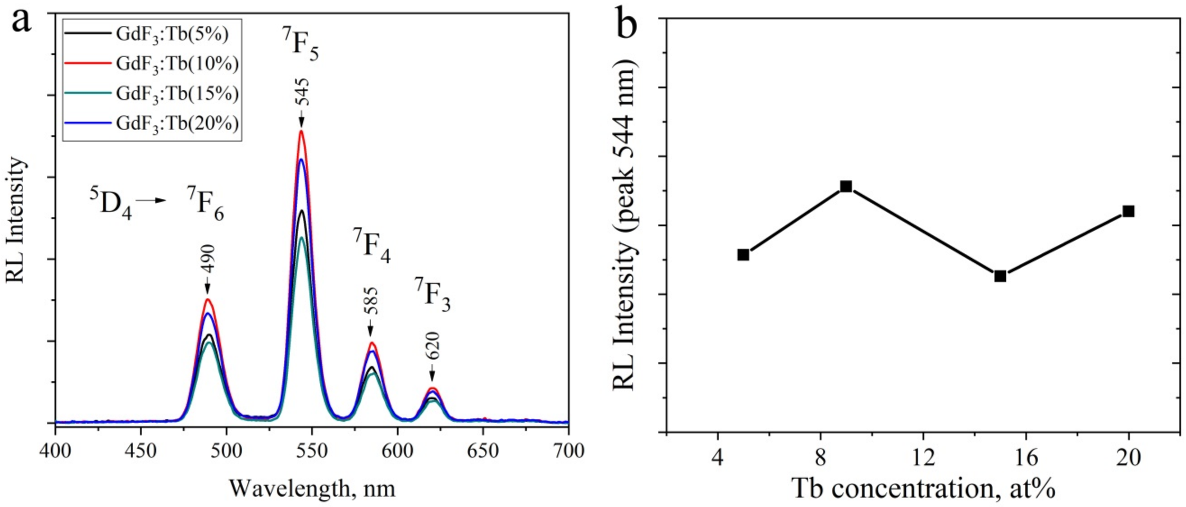

Figure S4. (a) Radioluminescence spectra of PEG@GdF_3: Tb^{3+}(x=5, 10, 15, 20%) nanoparticles excited by X-rays (35kV, 16mA). The main peaks localized at 490 nm, 544 nm, 585 nm and 620 nm, corresponding to electronic transitions from the excited states $^5D_4$ to the ground states ^7F_J (J = 6–3), (b) Dependence of the peak intensity at 545 nm of radioluminescence spectra of PEG@GdF_3: Tb^{3+}nanoparticles with different Tb^{3+} ion concentrations.
